# Filtration of Elastic Polymers and Spherical Gels through a Silica-Deposited Layer on a Porous Membrane

**DOI:** 10.3390/membranes11010022

**Published:** 2020-12-28

**Authors:** Takanori Hidane, Hidemi Kitani, Shintaro Morisada, Keisuke Ohto, Hidetaka Kawakita, Sachiko Furuta

**Affiliations:** 1 Department of Applied Chemistry, Saga University, Saga 840-8502, Japan; 18578028@edu.cc.saga-u.ac.jp (T.H.); 17578009@edu.cc.saga-u.ac.jp (H.K.); morisada@cc.saga-u.ac.jp (S.M.); ohtok@cc.saga-u.ac.jp (K.O.); 2 Saga Ceramics Research Laboratory, Saga 844-0022, Japan; furuta-sachiko@pref.saga.lg.jp

**Keywords:** filtration and adsorption, colloidal particle, elasticity, porous membrane, mathematical model

## Abstract

A 120-nm silica suspension was permeated through a porous polyethylene (PE) hollow-fiber membrane, as was a solution of deformable elastic particles of poly(*N*-isopropylacrylamide) (PNIPAM) gel and dextran. The amount adsorbed and flux of permeation were analyzed with ordinary differential equations to obtain adsorption coefficients, maximum amounts adsorbed, and pore-narrowing factors. The thickness of the “silica-deposited layer” on the membrane was 1 μm. In a batch adsorption mode, 5.0 mg of PNIPAM gel and 30 mg of dextran were adsorbed on the PE membrane, with no adsorption on the silica. The PE membrane pores were narrowed by a secondary layer of adsorbed PNIPAM gel. When filtered through the silica-deposited layer, PNIPAM gel occupies gaps, resulting in a reduced permeation flux. Dextran passed through the silica-deposited layer and was partially adsorbed on the PE membrane. The modified membrane can control adsorption, filtration, and flux permeation, which leads to dynamic membrane separations.

## 1. Introduction

Microfiltration has been used for the separation of cells and microorganisms as well as protein and polysaccharide separations. To enhance the separation efficiency, pore regulation was performed by introducing polymers into the membrane and by forming a skin layer of polymers and inorganic materials on the pore surface. These dynamic membranes thus have a layer of deposited particles and polymers on the pore surfaces [1]. Several deposited particles and polymers perform adsorption and filtration within the gaps of the layer materials. In particular, TiO_2_ and SiO_2_ particles have been deposited on membrane surfaces for water treatment and emulsion separations [2,3,4,5]. Deposited layers that enable adsorption and filtration can separate particles and molecules not only by size effects, but also by adhesion. 

Separation targets of microfiltration are rigid particles of silica and alumina with 50 nm–10 μm sizes, as well as deformable particles, such as microorganisms, gels, proteins, and linear polymers. Rigid particles would be filtered by membrane pores having the same sizes, while deformable particles could pass through the membrane or be filtered via depth filtration [6,7,8]. For the same size range, deformable particles are subject to fluid flow pressure and shear stress in the membrane, and thus pass through the pores via deformation [9,10]. Microchannel and track-etched membranes with narrowed cylindrical tubes have been studied for filtration permeabilities of deformable gels and polymers [11,12,13]. Poly(*N*-isopropylacrylamide) (PNIPAM) gels with one-tenth sizes relative to microchannel pores were passed through the pores to examine gel deformation [14]. 

Here, a suspension of 120-nm silica particles was permeated through a polyethylene (PE) microfiltration membrane to deposit a silica layer on the membrane pores. Then, a poly(*N*-isopropylacrylamide) gel and dextran solution (2000 kDa), with 50-nm particle sizes, was permeated through the membrane to examine the filtration and adsorption efficiency. Permeated silica particles form a silica layer on the membrane surface (creating a “silica-deposited” membrane). The filtration flux and the amount of adsorbed particles in the silica-deposited membrane was mathematically evaluated with Nakamura’s model [15,16]. A polarization model [17], a filtration resistance model [18], and a blocking model [19,20,21] have been studied. The novel mathematical model here includes adsorption coefficients on the silica-deposited layer and the PE membrane, and multilayer filtration by the PE membrane. The model quantifies the adsorption coefficients and the maximum amounts adsorbed to determine adsorption, filtration, and the filtration flux from the obtained parameters. 

Specifically, the steps in this study were as follows. (1) A silica layer was deposited on the PE membrane. (2) Solutions of deformable PNIPAM gel and linear dextran polymer (2000 kDa), with 50-nm sizes, were individually permeated to determine filtration fluxes and amounts adsorbed. (3) The filtrations of the two deformable particles were examined and analyzed using the proposed mathematical model. 

## 2. Theory

### 2.1. Calculation of Silica Layer Thickness

The total membrane resistance, *R_total_*, was calculated from the water-permeation flux through silica-deposited membrane, *J*_0_, at 0.05 MPa by Equation (1),

(1) $$R_{total}=\frac{1}{J_{0}}\frac{\Delta P}{\mu }$$

where Δ*P* and *µ* are the pressure drop and viscosity of pure water, respectively. The PE membrane resistance, *R_m_*, was also determined from the water flux, *J_m_*_0_, with Equation (2), and the resistance of the silica-deposited layer, *R_Si_*, was calculated from Equation (3).

(2) $$R_{m}=\frac{1}{J_{m0}}\frac{\Delta P}{\mu }$$

(3) $$R_{Si}=R_{total}-R_{m}$$

From Equations (4) and (5), the thickness of the silica-deposited layer, *L_Si_* was calculated with Equation (6).

(4) $$J_{Si}=N_{Si}A\frac{\pi r_{Si0}^{4}\Delta P_{Si}}{8\mu L_{Si}}$$

(5) $$R_{Si}=\frac{1}{J_{Si}}\frac{\Delta P_{Si}}{\mu }$$

(6) $$L_{Si}=\frac{1}{8}N_{Si}A\pi r_{Si}^{4}R_{Si}$$

*J_Si_*, *N_Si_*, *A*, *r_Si_*, and Δ*P_Si_* are the imaginary flux in the silica-deposited layer, the pore number density of the silica-deposited layer, the effective membrane area, the pore-gap radius in the silica-deposited layer, and the pressure drop in the silica-deposited layer, respectively. 

### 2.2. Mathematical Model of Filtration by the PE Membrane

**Figure 1:**
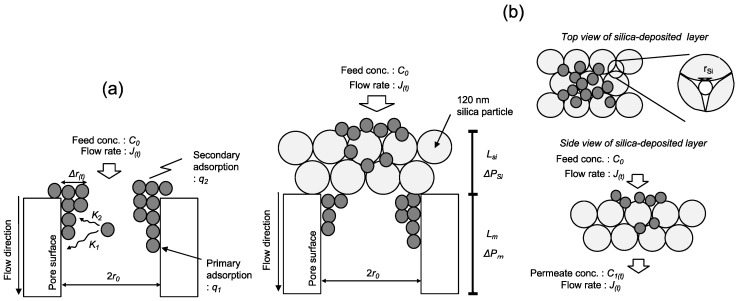
Schematic of filtration in a (**a**) PE membrane, and a (**b**) silica-deposited membrane.

A permeating colloidal suspension through a membrane can be filtered via adsorption and size exclusion, as shown in Figure 1a. The amounts adsorbed to the membrane pore surfaces and to previously adsorbed colloid surfaces are defined as *q*_1(*t*)_ and *q*_2(*t*)_, respectively. During colloid filtration, the membrane pore narrows, which results in increased membrane resistance. 

The water-permeation flux, *J_m_*_0_, through the PE membrane was determined at 0.05 MPa. The rates of colloid adsorption to the pore and previously adsorbed colloid surfaces are represented by Equations (7) and (8), respectively:

(7) $$\frac{dq_{1\left(t\right)}}{dt}=K_{1}C_{0}J_{\left(t\right)}A\left(1-\frac{q_{1\left(t\right)}}{Q_{max1}}\right)$$

(8) $$\frac{dq_{2\left(t\right)}}{dt}=K_{2}C_{0}J_{\left(t\right)}A\left(\frac{q_{1\left(t\right)}}{Q_{max1}}\right)\left(1-\frac{q_{2\left(t\right)}}{Q_{max2}}\right)$$

where *K*_1_, *J*_(*t*)_, *Q_max_*_1_, *K*_2_, and *Q_max_*_2_ are the adsorption coefficient on the membrane pore, the filtration flux, the maximum amount of colloid adsorbed, the adsorption coefficient on the previously adsorbed colloids, and the maximum amount on the adsorbed colloid, respectively. The initial concentration of the colloidal suspension is *C*_0_. This model assumes that (a) adsorption is irreversible, and that (b) multilayer adsorption occurs in a primary and secondary manner, with a certain maximum amount adsorbed.

Pore radius narrowing by the adsorbed colloid was calculated with Equation (9):

(9) $$r_{\left(t\right)}=r_{0}-\Delta r_{\left(t\right)}$$

where *r*_0_ and *Δr*_(*t*)_ are the initial pore radius and the adsorbed colloidal layer thickness, respectively.

(10) $$\Delta r_{\left(t\right)}=B_{1}q_{1\left(t\right)}+B_{2}q_{2\left(t\right)}$$

The latter was calculated with Equation (10). *B*_1_ and *B*_2_ are the thicknesses of the first and second layers, respectively, per unit amount of adsorbed colloid mass, a pore-narrowing factor. 

The flux with colloidal adsorption was determined with the Hagen-Poiseuille equation:

(11) $$J_{\left(t\right)}=N_{m}A\frac{\pi r_{\left(t\right)}^{4}\Delta P}{8\mu L_{m}}$$

where *N_m_* and *L_m_* are the pore density and thickness of the PE membrane, respectively.

Initial conditions were as follows:

(12) $$\;q_{1\left(t\right)}=q_{2\left(t\right)}=0\;\;\;\;\;\;\mathrm{at}\;t=0$$

(13) $$J_{\left(t\right)}=J_{0}\;\;\;\;\;\;\mathrm{at}\;t=0$$

Equations (7)−(11) were analyzed using the fourth-order Runge–Kutta method based on the initial conditions of Equations (12) and (13), with a time step of Δ*t* = 2 s. The parameters *K*_1_, *K*_2_, *B*_1_, *B*_2_, *Q_max_*_1_, and *Q_max_*_2_ are determined by curve fitting to the experimental data. 

### 2.3. Mathematical Model of Filtration by the Silica-Deposited Membrane

Filtration of the colloidal suspension by the silica-deposited layer and the PE membrane is depicted in Figure 1b. The amounts adsorbed on the silica-deposited layer, the PE membrane, and the previously adsorbed colloid are defined as *q_Si_*, *q*_1_, and *q*_2_, respectively. Silica did not adsorb the colloid in batch mode and the PNIPAM gel and dextran adsorbed only on the PE membrane pores. Filtration by the silica-deposited layer is related to the size-exclusion effect. The flux and *R_Si_*_0_ of that layer were defined as follows:

(14) $$J_{Si0}=N_{Si}A\frac{\pi r_{Si0}^{4}\Delta P_{Si}}{8\mu L_{Si}}$$

(15) $$R_{Si0}=\frac{1}{J_{Si0}}\frac{\Delta P_{Si}}{\mu }$$

where *N_Si_*, *r_Si_*_0_, *L_Si_*, and Δ*P_Si_* are the pore density, the pore radius, the thickness, and the pressure drop in the silica-deposited layer, respectively, and *ΔP_m_* is the pressure drop in the PE membrane. 

From Equations (14) and (15), *R_total_*_0_ was calculated by Equation (16). *N_Si_* was determined by fitting to:

(16) $$R_{total0}=R_{m0}+R_{Si0}$$

(17) $$J_{0}=\frac{1}{R_{total0}}\frac{\Delta P}{\mu }$$

*J*_0_ is calculated from Equation (17), where *R_Si_*_0_ and *R_m_*_0_ are the membrane resistances of the silica-deposited layer and PE membrane, respectively. Pressure drops created by the silica-deposited layer and the PE membrane are determined by Equations (18) and (19), respectively.

(18) $$\frac{\Delta P_{m}}{\Delta P_{Si}}=\left(\frac{L_{m}}{L_{Si}}\right)\left(\frac{N_{Si}}{N_{m}}\right){\left(\frac{r_{Si0}}{r_{0}}\right)}^{4}$$

(19) $$\Delta P=\Delta P_{Si}+\Delta P_{m}$$

The adsorption rate on the silica-deposited layer was determined by Equation (20):

(20) $$\frac{dq_{Si\left(t\right)}}{dt}=K_{Si}C_{1}J_{\left(t\right)}A\left(1-\frac{q_{Si\left(t\right)}}{Q_{maxSi}}\right)$$

where *K_Si_* and *Q_maxSi_* are the adsorption coefficient of the colloid on the silica-deposited layer and the maximum amount adsorbed, respectively. 

The pore radius in the silica-deposited layer, narrowed by the adsorbed colloid, was calculated with Equation (21). *B_Si_* is the thickness of the adsorbed colloid per adsorbed amount, and the filtration flux was defined by Equation (22). The total membrane resistance, *R_total_*, was defined by Equation (23), and *R_m_* and *R_Si_* were, respectively, from Equations (24) and (25):

(21) $$r_{Si\left(t\right)}=r_{Si0}-B_{Si}q_{Si\left(t\right)}$$

(22) $$J_{\left(t\right)}=\frac{1}{R_{total\left(t\right)}}\frac{\Delta P}{\mu }$$

(23) $$R_{total\left(t\right)}=R_{m\left(t\right)}+R_{Si\left(t\right)}$$

(24) $$R_{m\left(t\right)}=\frac{1}{J_{m\left(t\right)}}\frac{\Delta P_{m}}{\mu }$$

(25) $$R_{Si\left(t\right)}=\frac{1}{J_{Si\left(t\right)}}\frac{\Delta P_{Si}}{\mu }$$

*J_Si_*_(*t*)_ and *J_m_*_(*t*)_ are the imaginary fluxes in the silica-deposited layer and the PE membrane, respectively, and are defined by Equations (26) and (27), respectively.

(26) $$J_{Si\left(t\right)}=N_{Si}A\frac{\pi r_{Si\left(t\right)}^{4}\Delta P_{Si}}{8\mu L_{Si}}$$

(27) $$\;J_{m\left(t\right)}=N_{m}A\frac{\pi r_{\left(t\right)}^{4}\Delta P_{m}}{8\mu L_{m}}$$

Equations (7)–(11) and (14)–(27) were analyzed using the fourth-order Runge–Kutta method based on the following initial conditions of Equations (28) and (29), with time step *Δt* = 2 s to obtain the *K_Si_*, *Q_maxSi_*, and *B_Si_* parameters by fitting the experimental data. *K*_1_, *K*_2_, *B*_1_, *B*_2_, *Q_max_*_1_, and *Q_max_*_2_ were used from the PE membrane.

(28) $$q_{1\left(t\right)}=q_{2\left(t\right)}=q_{Si\left(t\right)}=0\;\;\;\;\;\;\mathrm{at}\;t=0$$

(29) $$J_{\left(t\right)}=J_{1}\;\;\;\;\;\;\mathrm{at}\;t=0$$

## 3. Experiments

### 3.1. Materials

**Figure A1:**
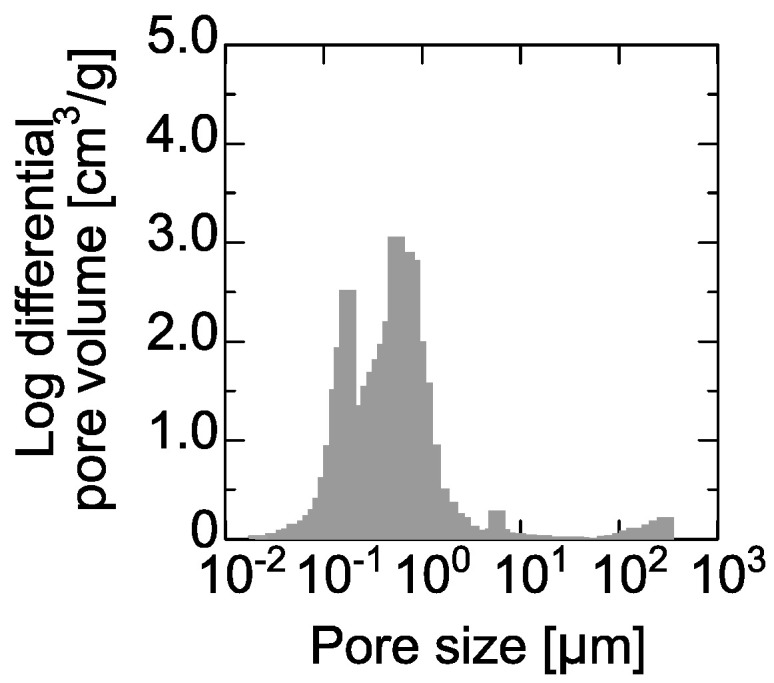
Pore distribution of PE membrane.

Porous hollow-fiber PE membranes were obtained from Asahi Kasei Corporation, Tokyo. The inner and outer diameters were 2.0 mm and 3.0 mm, respectively. The pore size distribution was determined by mercury intrusion, as shown in the Appendix A (Figure A1), and had peaks at 100 nm and 360 nm. The porosity of the PE membrane was 71%. Spherical silica particles (120-nm and 50-nm mean size) were obtained from JGC Catalysts and Chemicals Ltd., Kawasaki, Japan. *N*-isopropylacrylamide (NIPAM), *N*,*N*-dimethylacrylamide (DMAM), *N*,*N*′-methylene bisacrylamide (BIS), *N*,*N*,*N*′,*N*′-tetramethyl ethylenediamine (TEMED), sodium dodecyl sulfate (SDS), ammonium persulfate (APS), and dextran (2000 kDa) were obtained from Sigma-Aldrich Co. LLC., Tokyo, Japan. Regenerated cellulose membranes (MWCO 50 kDa) were obtained from Fujifilm Wako Pure Chemical Corporation, Japan. Other chemicals were of analytical grade or higher.

### 3.2. Preparation of PNIPAM Gel

NIPAM (0.36 M), DMAM (0.04 M), BIS (0.04 M), and TEMED (0.012 M) were mixed in a three-necked flask and solubilized in pure water. The SDS (0.01 M) and APS (0.001 M) solutions were individually prepared. The monomer solution (50 mL) was bubbled with nitrogen gas and mixed at 250 rpm, and the SDS solution was added with a syringe at 333 K. After 30 min, the APS solution was added to start the polymerization. To remove SDS from the PNIPAM gel solution after the polymerization, the solution was dialyzed with a regenerated cellulose membrane for a week. The PNIPAM gel was analyzed using electrophoretic light scattering (ELSZ-2Plus, Otsuka Electronics Co., Ltd., Japan). 

### 3.3. Batch-Mode Adsorption of PNIPAM Gel and Dextran to the PE Membrane and 120-nm Silica 

The concentration of the PNIPAM gel suspension (0.5 mL) was set in the range of 0.01–0.64 g/L. It was then was mixed with the silica suspension (0.5 mL) or a 1.0-cm PE membrane at 303 K for 200 h at neutral pH. After the adsorption, the concentration that remained in solution was determined by ultraviolet-visible absorption (UV-1800, Shimadzu, Japan) at 260 nm. 

The concentration of the dextran solution (0.5 mL) was set over the range 0.10–1.0 g/L, and was mixed with the silica suspension (0.5 mL) or the PE membrane (1.0 cm) for adsorption at 303 K for 200 hours at neutral pH. After the adsorption, the remaining concentration of dextran was determined by the phenol-sulfuric acid method.

### 3.4. Deposition of Silica on the PE Membrane

The permeation equipment included a peristatic pump (IWAKI Co., Ltd., Tokyo), a pressure gauge (Nagano Keiki Co., Tokyo, Japan), and a porous hollow-fiber PE membrane (5.0 cm effective length). The silica-deposited membrane was prepared in the following sequence: (1) ethanol and water were permeated through the PE membrane for hydrophilization of the pores; (2) the 120-nm silica suspension (0.040 g/L) was then permeated through the membrane at a constant pressure of 0.05 MPa; and (3) by opening a three-way cock valve from the dead-end mode, water was allowed to flow at 600 mL/h for 5.0 min through the lumen part of the membrane. The modified membrane was referred to as the “silica-deposited membrane.” The pore surface on the lumen was observed by scanning electron microscopy (SU-1500, Hitachi Ltd., Japan). The amount of silica adsorbed on the membrane was determined from its concentration before and after the permeation process. 

### 3.5. Permeation of Colloidal Suspension through the Membrane

The same permeation equipment was used to permeate the PNIPAM gel suspension (0.3 g/L) and the dextran solution (0.1 g/L) through the PE and the silica-deposited membranes at room temperature and constant 0.05-MPa pressure. The concentration of the PNIPAM gel and dextran in the effluent collected from the membranes were determined by absorption at 260 nm and the phenol-sulfuric acid method, respectively. The filtration flux and the amount of adsorbed material were defined by Equations (30) and (31):

Filtration flux, *J*, [m/h] = (volume of effluent from membrane)/(effective surface area) (30)

Amount adsorbed [g] = Σ(*C*_0_ − *C_i_*)*V_i_* (31)

where *C*_0_, *C_i_*, and *V_i_* are the initial colloid concentration, the concentration in each fraction, and the volume of effluent, respectively.

## 4. Results

### 4.1. Silica Deposition on PE Membrane Surface

The 120-nm silica suspension was permeated through the PE membrane for 50 min to form the silica-deposited layer on the membrane pores. This layer would function as a new separation and filtration domain. As noted above, the pore distribution of the PE membrane had peaks at 100 nm and 360 nm. During permeation of the silica suspension in the PE membrane, no silica was determined in the effluent. Therefore, all the silica remained in or on the PE membrane. 

**Figure 2:**
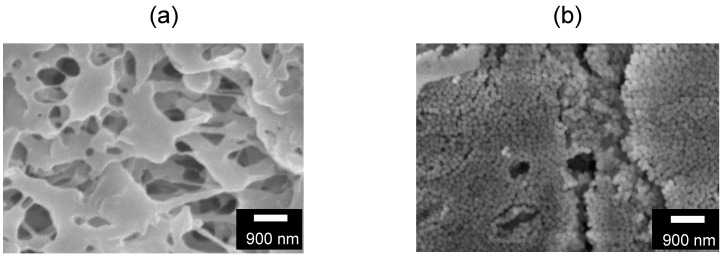
Scanning electron microscopy images of pore surfaces of the (**a**) PE membrane and the (**b**) silica-deposited membrane.

Scanning electron microscope images of the pore surfaces of the PE and silica-deposited membranes are shown in Figure 2. As shown in Figure 2b, the silica-deposited membrane had a packed silica layer on top of the pores, and was not deposited in a depth-filtration manner. The deposited silica thickness, *L_Si_*, was determined by Equations (1)–(6), and the values used in the calculations are summarized in Table 1. That is, the values in Table 1 were inserted in Equation (6) to obtain the 0.96-μm thickness of the silica-deposited layer and to show the tenth layer of the 120-nm silica. 

### 4.2. Adsorption of PNIPAM Gel and Dextran on the PE Membrane and 120-nm Silica 

**Figure A2:**
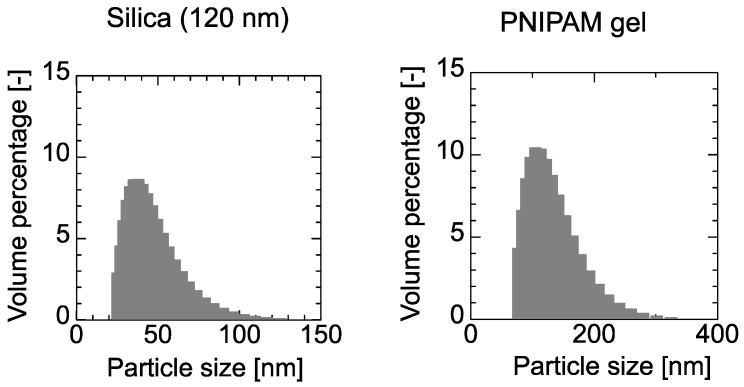
Supporting information, size distribution of silica and PNIPAM gel.

The size distributions of the 120-nm silica and the PNIPAM gel were determined by dynamic light scattering, as shown in the Appendix A (Figure A2). Dextran (2000 kDa) had a hydrodynamic diameter of 50 nm, as determined by the Einstein-Stokes equation [22]. Therefore, the PNIPAM gel and the dextran had the same diameters, but with different flexibilities.

**Figure 3:**
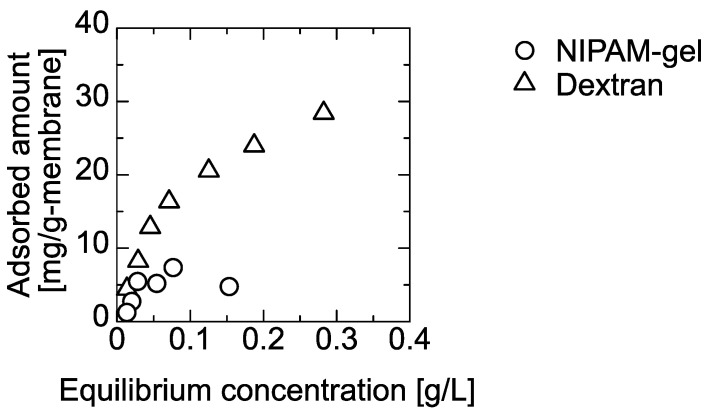
Batch mode adsorption of PNIPAM gel and dextran on the PE membrane at 303 K.

The PNIPAM gel and dextran solution was permeated through the silica-deposited and PE membranes for filtration and adsorption. In the batch mode, the adsorption of the PNIPAM gel and the dextran on the PE membrane were determined at 303 K, and the amounts adsorbed as a function of equilibrium concentration are shown in Figure 3. The adsorbed amount increased with the equilibrium concentration and leveled off. The maximum amounts adsorbed were 30 mg PNIPAM gel and 5.0 mg of dextran per 1 g of PE membrane. The 120-nm silica did not adsorb the PNIPAM gel and dextran because it had hydroxyl groups on the surface. Dextran is a flexible linear polymer with α-(1,6) glucose binding and a random linear structure. Conversely, PNIPAM gel is a cross-linked polymer with a more rigid structure. Because the adsorption was performed at 303 K, the PNIPAM gel was hydrated to form more hydrophilic characteristics [23]. The dextran was predicted to have a hydrophobic interaction with the PE membrane because of its flexible structure.

### 4.3. Filtration of PNIPAM Gel and Dextran through the Membrane

**Figure 4:**
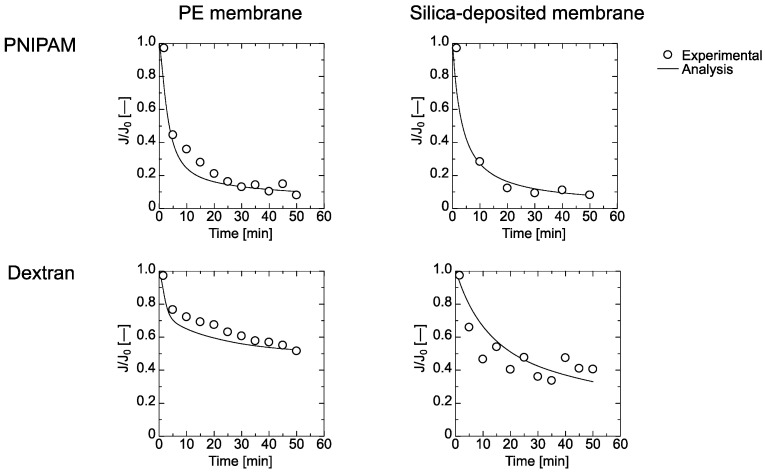
Flux time course curves of PNIPAM gel and dextran solutions through PE and silica-deposited membranes.

**Figure 5:**
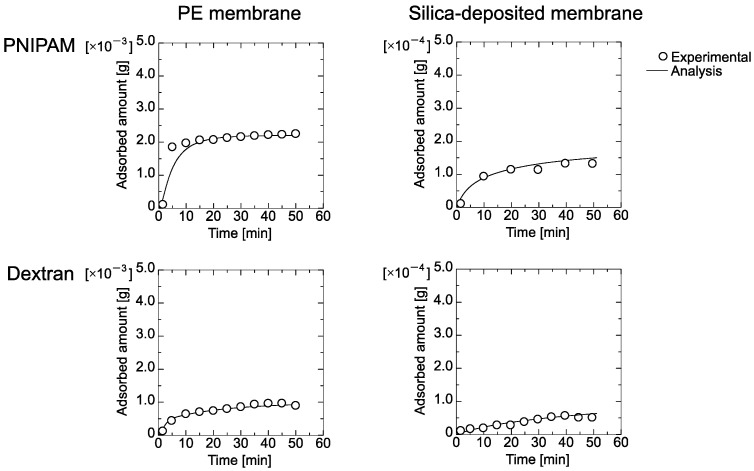
Time course curves of adsorbed amount of PNIPAM gel and dextran through PE and silica-deposited membranes.

Time course curves for the flux and the amount of adsorbed PNIPAM gel and dextran on the PE and the silica-deposited membranes are shown in Figure 4 and Figure 5, respectively. The flux through both membranes was reduced by filtration of the particles. The flux of the PNIPAM gel declined more than that of the dextran. The flux through the silica-deposited membrane declined more quickly than that of the PE membrane, which indicated that the silica worked as a dynamic filtration layer. 

Figure 5 shows that the amount adsorbed by the PE membrane was higher than that of the silica-deposited membrane. This was because the PE membrane exhibited a higher flux that transported particles to the inner part of the membrane, which is opposite to the batch-mode adsorption shown in Figure 3. Nakamura and Matsumoto reported that static adsorption was different from dynamic adsorption because the convective flow through the pores transported the colloidal particles to the inner region that enhanced the amount adsorbed [16]. Pressure and shear stress during dextran permeation via convection through the PE membrane deformed the particles [10,24], which resulted in reduced adsorption. Because the silica-deposited layer governs the filtration performance by size-exclusion, the amount of adsorbed PNIPAM gel and dextran decreased. 

The experimental data in Figure 4 and Figure 5 were fitted by Equations (7)–(29). The obtained parameters for the PE and silica-deposited membranes are summarized in Table 2 and Table 3, respectively. *K*_1_ for the PNIPAM gel had a higher value than that of dextran, which indicates that dextran was adsorbed more by the PE membrane. However, *K*_2_ for dextran was lower than that for the PNIPAM gel, which is greater in the second layer. The maximum amount of adsorbed dextran on the first layer, *Q_max_*_1_, was higher than *Q_max_*_2_, but that of the PNIPAM gel on the second layer, *Q_max_*_2_, was higher than *Q_max_*_1_, which demonstrates that PNIMAM gel forms more multilayers. The pore-narrowing factors *B*_1_ and *B*_2_ of the PNIPAM gel were higher due to less PNIPAM gel deformation than for dextran, which resulted in occupation of the membrane pores. 

The maximum amount adsorbed, *Q_maxSi_*, and *K* indicated that the less deformable PNIPAM gel was filtered by the silica-deposited layer. This was because batch-mode adsorption of PNIPAM gel on the 120-nm silica did not occur. However, the pore-narrowing factor *B_Si_* of PNIPAM gel on the silica-deposited layer was comparable to that of dextran. 

## 5. Discussion

Silica and polymer were deposited on membrane pores to introduce a new dynamic functionality for microfiltration, ultrafiltration, and nanofiltration. The separation and filtration performance were controllable by the materials in the dynamic layer. 

Here, a 120-nm silica suspension was permeated through a PE membrane to deposit a silica layer to filter 50-nm-diameter PNIPAM gel and dextran particles. The PNIPAM gel is hydrated at room temperature. Dextran also has many hydroxyl groups, but it deforms with flexibility because it is a linear polymer. PNIPAM gel and dextran did not adsorb to the silica in batch mode. Thus, the silica-deposited layer only works for filtration. The amount of dextran adsorbed on the PE membrane was higher than that of the PNIPAM gel (Figure 3). This was because the fifth carbon on the dextran glucose ring would be relatively un-hydrated to possibly interact hydrophobically with the PE membrane. With the change of the applied pressure, the deformable particle was filtered by the pressure in the membrane. With increasing filtration time, the amount of filtered would be changeable. 

**Figure A3:**
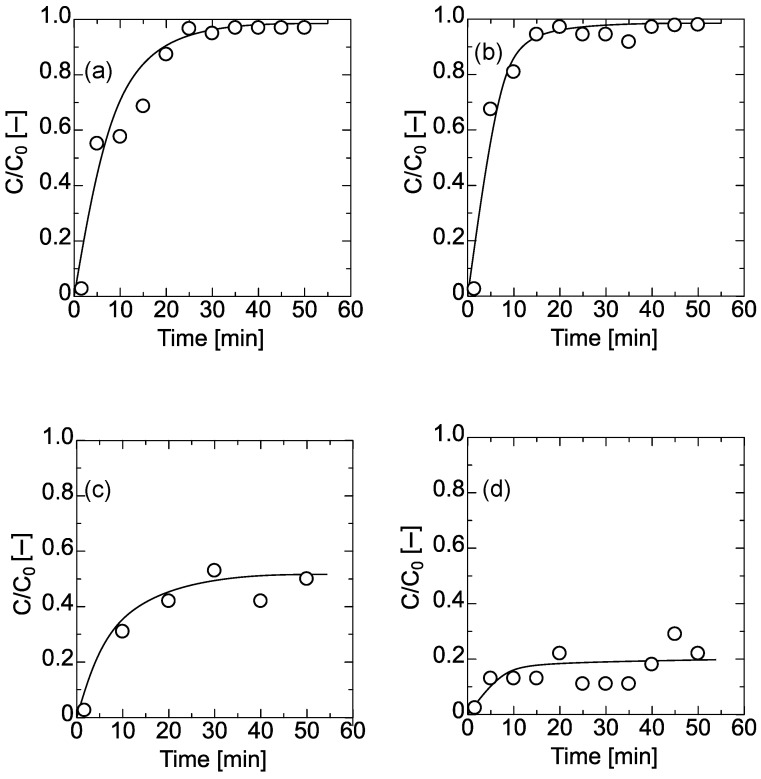
Concentration ratio in effluent per initial concentration as a filtration time. (**a**) PE mem-brane for PNIPAM gel, (**b**) PE membrane for dextran, (**c**) silica-deposited membrane for PNIPAM gel, and (**d**) silica deposited membrane for dextran.

Figure 5 shows that the amount adsorbed by the PE membrane was higher than that of the silica-deposited membrane. This was because the PE membrane exhibited a higher flux that transported particles to the inner part of the membrane, which is opposite to the batch-mode adsorption shown in Figure 3. The retention coefficient to PE membrane and silica-deposited membrane was already evaluated, as shown in Appendix A (Figure A3).

PNIPAM gel and dextran deform by external applied forces. When their solutions were flowed through the silica-deposited membrane, they were deformed by the applied permeation pressure and the shear stress of the gaps in the cake-deposited layer and the PE membrane pores. Because dextran is not cross-linked, its degree of deformation is higher. Marszalek et al. [25] and Neelov et al. [26] used molecular dynamics calculations and atomic force microscopy to determine that dextran deforms under forces greater than 100 pN. Defining the viscosity of solution, the filtration flux, and the membrane pore radius *μ* Pa·s, *J* m·s^−1^, and *r*_PE_ m, respectively, the applied shear stress to dextran adsorbed on the membrane was easily defined as follows,

*τ* = *μ* (Δ*J*)/(Δ*r*_PE_ ) (32)

Insertion of *μ* Pa·s, *J* m·s^−1^, and *r*_PE_ m, values of 1 mPa·s, 1.0 ×10^−2^ m·s^−1^, and 180 nm, respectively, to the above equation, yields a *τ* of 0.1 Pa. This pressure deforms dextran, and filtered dextran on the silica-deposited layer deformed more than the PNIPAM gel. Some dextran would go through the PE membrane, while the PNIPAM gel occupied the silica-deposited layer pores because it was less deformed. 

**Figure 6:**
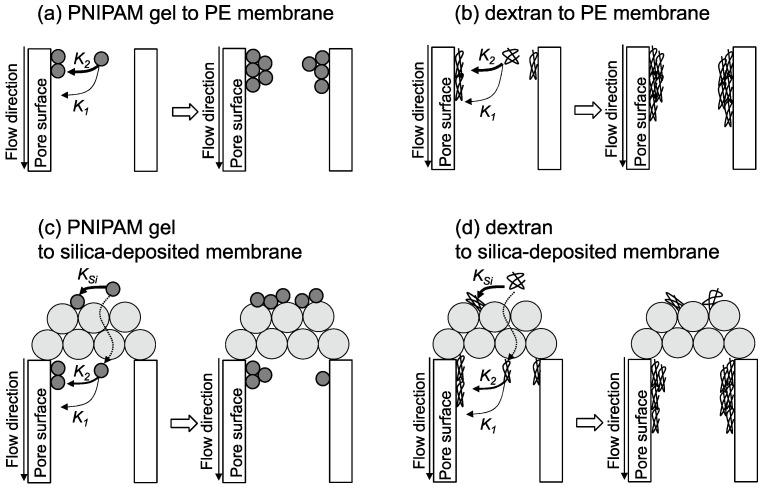
Schematics of PNIPAM gel and dextran filtration by PE and silica-deposited membranes. (**a**) permeation of PNIPAM gel solution to PE membrane, (**b**) permeation of dextran solution to PE membrane, (**c**) permeation of PNIPAM gel solution to silica-deposited membrane, and (**d**) permeation of dextran solution to silica-deposited membrane.

Based on the above analysis, schematics of PNIPAM gel and dextran filtered by the PE and silica-deposited membranes are shown in Fig. 6. In the case of filtering by the PE membrane (Figure 6a,b), linear dextran adsorbs to the pore surfaces by multi-point interaction. PNIPAM gel filtered by the size-exclusion effect could occupy the pores and the interaction with the pores occurs via entanglement because the *K*_2_ of PNIPAM gel had a high value. In the case of filtering by the silica-deposited membrane (Figure 6c,d), the PNIPAM gel would be primarily filtered by the silica-deposited layer, but the dextran would pass through because of deformation of the globule to a linear structure. Some dextran would be adsorbed by the PE membrane. Dextran filtered by the silica-deposited layer would fill gaps in the layer and reduce the flux. Nanoparticle at the smaller size could be deposited on the membrane with the smaller size, such as ultrafiltration. The analysis proposed by this study is applicable layer of the modified silica can be used for the separation and reaction media.

## 6. Conclusions

A silica-deposited membrane was prepared by permeating a silica suspension through a PE membrane. It was used to filter deformable PNIPAM gel and dextran particles. The flux and the amount of adsorbed particles were analyzed with ordinary differential equations to quantify the adsorption, filtration, and occupation of the membrane pores. Each particle would be adsorbed in the pores to reduce the flux, and dextran would deform in the pore to adsorb in a multi-point interaction. The silica-deposited membrane filtered PNIPAM gel via the size-exclusion effect, which resulted in a reduced flux and occupation of gaps in the silica layer. This layer on a commercially available membrane modified with various colloidal particles and polymers improves the filtration performance. Colloids having the same size deform via shear stress and applied pressure to enable filtration through the narrowed pores. Conventionally, the deposited layer on the membrane surface had a drawback due to serious fouling. However, when the deposited silica works as a new adsorption site, the filtration as well as the adsorption would appear. These roles are quantitatively evaluated from the proposed mathematical analysis, leading to the potential separation by the membrane.

## Figures and Tables

**Table 1:** Properties of silica-deposited layer.

pore number density of silica deposited layer, *N_Si_* [m^−2^]	4.1 × 10^16^
effective membrane area, *A* [m^2^]	3.1 × 10^−4^
pore-gap radius in silica deposited layer, *r_Si_* [m]	1.7 × 10^−8^

**Table 2:** Calculated fitting parameters to PE membrane.

	*K*_1_ (m^−1^)	*K*_2_ (m^−1^)	*Q_max_*_1_ (g)	*Q_max_*_2_ (g)	*B*_1_ (m/g)	*B*_2_ (m/g)
PNIPAM	1.0 × 10^−3^	1.5 × 10^0^	8.0 × 10^−5^	2.2 × 10^−3^	1.0 × 10^−3^	2.0 × 10^−5^
Dextran	1.0 × 10^−2^	5.0 × 10^−1^	6.0 × 10^−4^	3.7 × 10^−4^	3.0 × 10^−5^	3.0 × 10^−5^

**Table 3:** Calculated parameters to silica-deposited membrane.

	*K*_1_ (m^−1^)	*K_2_* (m^−1^)	*Q_max_*_1_ (g)
PNIPAM	1.7 × 10^−1^	2.0 × 10^−4^	5.5 × 10^−4^
Dextran	2.3 × 10^−2^	3.0 × 10^−5^	1.3 × 10^−3^

## Nomenclature

*A*	effective membrane area, m^2^
*B*_1_	thickness of first layer per unit amount of adsorbed colloid mass, pore-narrowing factor, m/g
*B*_2_	thickness of second layer per unit amount of adsorbed colloid mass, pore-narrowing factor, m/g
*B_Si_*	thickness of adsorbed colloid per amount of adsorbed colloid in silica-deposited layer, m/g
*C*_0_	initial concentration of colloidal suspension, g/m^3^
*C_i_*	concentration of each fraction from the membrane, g/m^3^
*J*_0_	water-permeation flux of silica-deposited membrane, m/s
*J_m_*_0_	water flux through PE membrane, m/s
*J_Si_*	imaginary flux through silica-deposited layer, m/s
*J*(*t*)	filtration flux, m/s
*K*_1_	adsorption coefficient on the membrane pore, 1/m
*K*_2_	adsorption coefficient on the adsorbed colloids, 1/m
*K_Si_*	adsorption coefficient of colloid on the silica-deposited layer, 1/m
*L_m_*	thickness of PE membrane, m
*L_Si_*	thickness of silica-deposited layer, m
*N_m_*	pore density of PE membrane, 1/m^2^
*N_Si_*	pore number density of silica-deposited layer, 1/m^2^
Δ*P*	pressure drop, Pa
Δ*P_m_*	pressure drop in PE membrane, Pa
Δ*P_Si_*	pressure drop in silica-deposited layer, Pa
*q*_1_(*t*)	amounts adsorbed to the membrane pore surface, g
*q*_2_(*t*)	amounts adsorbed to the previously adsorbed surface of the colloids, g
*Q_max_*_1_	maximum amount of colloid adsorbed, g
*Q_max_*_2_	maximum amount to the previously adsorbed colloid, g
*Q_maxSi_*	maximum amount adsorbed to the silica-deposited layer, g
*r_PE_*	radius of pore of PE membrane, m
*r_Si_*	pore-gap radius in silica-deposited layer, m
*R_m_*	PE membrane resistance, 1/m
*R_Si_*	resistance of silica-deposited layer, 1/m
*R_total_*	total membrane resistance, 1/m
*V_i_*	volume of effluent from the membrane, m^3^
*µ*	viscosity, Pa·s
